# Women's Perspectives on a Reproductive Health Services Screening Question: An Alternative to Pregnancy Intention Screening

**DOI:** 10.1089/whr.2022.0068

**Published:** 2022-11-28

**Authors:** Silpa Srinivasulu, Meredith G. Manze, Heidi E. Jones

**Affiliations:** ^1^ Department of Community Health and Health Policy, City University of New York Graduate School of Public Health and Health Policy, New York, New York, USA.; ^2^ Department of Epidemiology and Biostatistics, City University of New York Graduate School of Public Health and Health Policy, New York, New York, USA.; ^3^ The CUNY Institute for Implementation Science in Population Health, New York, New York, USA.

**Keywords:** contraception, preconception, pregnancy intentions, primary care, qualitative, reproductive health

## Abstract

**Background:**

Current efforts to integrate reproductive health care into primary care in the United States involve assessing pregnancy intentions and reproductive goals, which are often not meaningful or attainable for some. Alternatively, we designed a reproductive health services-based screening question: “Can I help you with any reproductive health services today, such as preventing pregnancy or planning a healthy pregnancy?” In this study, we describe women's interpretations of this question as part of a larger study, exploring perspectives on reproductive health care quality in primary care.

**Materials and Methods:**

We utilized a third-party research firm to recruit New York women of reproductive age (18–45), who visited a primary care provider in the past year. We conducted five focus groups and eight interviews (*N* = 30). Semistructured guides queried participants on interpretations of the screening question and preferences for raising reproductive health concerns during a primary care visit. We employed inductive thematic analysis.

**Results:**

Participants interpreted the question as offering contraception or pregnancy counseling and care, although younger participants also understood it as offering sexual and reproductive health services broadly. Participants also connected the question with discussions about their ability to conceive. Some participants described experiences with provider assumptions and implicit bias. Tensions emerged around accepting primary care as a setting for reproductive health due to a perceived lack of specialized training.

**Conclusions:**

Participants interpreted the screening question as intended, indicating face validity. Primary care settings should increase patients' awareness of reproductive health service availability, such as by routinely introducing a services-based screening question.

## Introduction

Providing high-quality, comprehensive, patient-centered sexual and reproductive health (SRH) services in primary care is critical for improving SRH outcomes. To this end, screening initiatives using pregnancy intention and reproductive life planning frameworks to enhance preconception and contraception care have been adopted by many primary care practices and endorsed by US public health and professional organizations.^1–4^ Reproductive life planning is a counseling strategy in which women are encouraged to identify their reproductive goals and pregnancy intentions, in the context of their personal values and goals, and make a life plan.^3,5,6^ Pregnancy intention screening with One Key Question^®^ refers to asking patients “would you like to become pregnant in the next year?” to assess patients' desires to achieve or avoid pregnancy.^7^

While both frameworks are designed to increase access to SRH services, they are not always salient, as many women do not possess unequivocal, binary pregnancy intentions and may have complex, ambivalent feelings that fall along a spectrum.^6,8–11^ Pregnancy attitudes, plans, and emotions tend to inform patients' service needs. Current approaches rely on time- and goal-oriented questions regarding pregnancy intention and plans.^12^ A planning-based paradigm may portray implicit assumptions about mistimed or unwanted pregnancies being inherently negative and causing adverse outcomes.^10^ Multiple articles describe these conceptual flaws in measuring and utilizing “unintended” pregnancy as a public health indicator, including both abortions and unintended births as equivalent outcomes and categorizing both as inherently adverse; contradictions in how survey respondents answer questions on unintended pregnancy; differences in intention, wantedness, and timing of pregnancy; and its oversimplification of complexities surrounding pregnancy decisions and unique social contexts in which pregnancies occur.^13,14^

In addition, systematic reviews and studies have found an inconclusive effect of intentions screening on contraceptive use and pregnancy-related health outcomes.^15–19^ Taken together, discussing pregnancy “intentions” and reproductive life “plans” in the clinical setting is often not meaningful and may be perceived as disrespectful by some patients.

To move away from the “planning” paradigm, our group developed a services-based screening question born out of key informant interviews with diverse stakeholders, including family physicians, obstetrician/gynecologists (Ob/Gyns), health educators, advocates, and public health professionals: “Can I help you with any reproductive health services today, such as preventing pregnancy or planning a healthy pregnancy?”^20^ We found utilizing this question in primary care was considered feasible, acceptable, and preferable over other questions by patients and providers in New York, due to its inclusiveness, open-endedness, and promotion of reproductive autonomy.^21–23^ Patients reported feeling comfortable with this question and that it demonstrated providers cared about them.^21,23^ Providers also perceived it as an easy way to start a conversation on SRH.^22^

Studies have found that many primary care providers (PCPs) do not routinely screen for SRH needs, provide preconception or contraceptive counseling, or discuss sexual health.^22,24,25^ By asking broadly about service need, rather than pregnancy intentions, the question can be asked to patients of all ages, genders, sexual orientations, and backgrounds, without preconceived notions of who may be “at risk” for pregnancy. Therefore, this question embodies principles of patient-centeredness in that it is respectful of and responsive to individual patient preferences, needs, and values.^26^ This screening question was integrated into practice and tested for feasibility and outcomes within a large Federally Qualified Health Center network in New York City and resulted in a modest 3.4% average adjusted increase in the documentation of family planning services, including counseling, across seven sites, with more work needed to improve implementation equally across sites.^27,28^

Although many patients find this question acceptable and preferable to other reproductive health screenings, how they interpret this question remains unknown.^23^ Understanding how patients interpret this question is critical before widespread implementation in US primary care systems. To deepen our understanding, we queried New York State women on their interpretation of this question as part of a larger study on metrics for measuring quality of reproductive health care in primary care settings.

## Materials and Methods

### Study design and data collection

We conducted a qualitative study involving semistructured virtual focus groups and interviews with a purposive sample of women of reproductive age between October and December 2021. Eligibility criteria included women 18–45 years of age, currently living in New York State, who spoke and read English, self-identified as women, and had seen a PCP in the past year. We only recruited women of reproductive age; separate studies with people of other genders and ages are needed to understand their unique experiences and perceptions. We utilized a third-party research firm to recruit. They administered our survey screener, which queried on the above eligibility criteria, described the study, and invited participants to share their contact information.^29^

The firm established quotas to recruit 80 respondents within each age group: 18–25, 26–35, and 36–45. We contacted survey respondents in waves within each age group to invite them to prescheduled virtual focus groups. We aimed to conduct six focus groups, two per age group, with 5–6 participants per group. We planned to schedule 8–10 participants per group to allow for attrition. We emailed those who expressed interest in informed consent document describing the study, videoconferencing log-in information, and guidance on changing display names to chosen pseudonyms.

We conducted focus groups to allow group interactions to reveal how participants perceive and experience SRH. Group interaction enables participants to present their own views, hear, question, and comment on others’, and engage in continued discussion.^30^ The semistructured guide included questions on experiences and perceptions of optimal primary care visits for reproductive health care, as the principal study aim explored patient perspectives on measurement of reproductive health care quality.

As a secondary aim, we asked them to describe what they thought their PCP would mean if they were asked during their next primary care visit, “can I help you with any reproductive health services today, such as preventing pregnancy or planning for a healthy pregnancy?” We further probed participants on their preferences for raising SRH concerns during an appointment with their PCP, even if the appointment was for another health issue. In this study, we focus our analysis on these secondary aims.

Focus groups lasted 50 minutes to 2 hours. We discussed the informed consent document and obtained verbal consent from each participant before audio-recording. Immediately after each focus group, the co-moderators (M.G.M. and S.S.) and principal investigator (H.E.J.) practiced reflexivity by discussing emergent themes and patterns, group dynamics, strategies to improve moderation, potential revisions to the focus group guide, and personal reflections. Reflecting on ideas, processes, and experiences as they occur allows researchers to improve data collection, uncover more about themselves and their topic, including how assumptions may impact qualitative inquiry, and deepen analysis.^31^

Due to scheduling and participation challenges with age groups 18–25 and 26–35, we transitioned to in-depth interviews with these age groups to alleviate scheduling challenges and the potential discomfort participants may have in discussing SRH with strangers. We adapted the focus group guide for interviews, and we followed the same procedures for scheduling, informed consent, and data collection for interviews as we did focus groups. Interviews lasted from 25 to 60 minutes.

Immediately after each focus group and interview, we emailed participants a brief demographic survey. Participants received $30 for participating within 24 hours of completing the survey. We also captured concurrent field notes, reflecting on methodological considerations, subjectivities, and emergent patterns. Audio recordings were transcribed by a third-party professional service; only participant-selected pseudonyms were used for on-screen names, in transcriptions, and reported here. The Institutional Review Board of the City University of New York approved this study (Protocol No. 2021-2006-PHHP).

### Analysis

We employed inductive thematic analysis to explore participants' interpretations of the screening question and preferences for SRH discussions with PCPs.^32^ The analysts (M.G.M. and S.S.) identify as cis women and reproductive health researchers, one with and one without a child. Both believe SRH services should be accessible in primary care. We independently read the first three focus group transcripts (one from each age group), took margin notes, employed block coding, and reviewed field notes. We created an initial codebook through this process. We reviewed coding, reconciled discrepancies, and refined the codebook. One analyst (S.S.) coded all remaining transcripts and reviewed coding questions with the team. M.G.M. served as a second coder for interviews she did not conduct to ensure familiarity with all data.

Throughout the coding process, we practiced reflexivity and wrote memos to document patterns, connections to literature, and emerging constructs. We used field notes and memos first to identify themes and generate initial theory with respect to the research question. Then, we sorted excerpts with specific codes related to provider types, the screening question, and reproductive health history using a data display to deepen analysis. We utilized Dedoose version 9.0.17 (Los Angeles, CA) to manage data. We did not find differences in findings between focus group and interview respondents, so we present combined results.

## Results

We collected data from 22 participants in five focus groups and from 8 participants through in-depth interviews, for a total of 30 women of reproductive age (Table 1). We present participants' understanding of the reproductive health services screening question, “can I help you with any reproductive health services today, such as preventing pregnancy or planning for a healthy pregnancy?” Then, we discuss two additional themes regarding their interpretations: risk and history of provider bias on services offered, and tensions regarding primary care and specialty settings for reproductive health.

**Table 1.** Demographic Characteristics of Focus Group and Interview Participants (*N* = 30)

Characteristic	***n*** (%)
Age group	
18–25	10 (33.3)
26–35	8 (26.7)
36–45	12 (40)
Location in New York State	
New York City based (5 boroughs)	18 (60)
Outside of New York City	12 (40)
Race/ethnicity	
White	12 (40)
Black/African American	9 (30)
Asian	4 (13.3)
Hispanic/Latino/Spanish	4 (13.3)
American Indian/Alaskan Native	1 (3.3)
Health insurance status (*n* = 28)	
Private	14 (46.7)
Public	14 (46.7)
Feels they have regular health care provider^a^	25 (83.3)
Education	
College graduate or higher	14 (46.7)
Some college	9 (30)
High school graduate, no college	6 (20)
Some high school	1 (3.3)
Relationship status	
Single	15 (50)
Married or committed relationship	14 (46.7)
Divorced/separated/widowed	1 (3.3)
Sexuality (*n* = 29)	
Sex with men	20 (67)
Sex with women	6 (20)
Sex with men and women	3 (10)
Ever used some form of hormonal contraception	23 (77)
Self-reported ability to get pregnant^b^	
Yes	18 (60)
Unable	3 (10)
Unsure	9 (30)

^a^ We asked participants: “do you feel like you have a regular health care provider?” to assess whether participants think they have a usual provider to go to for health care.

^b^ We asked participants: “are you physically able to get pregnant?” as some may know about sterilization or fertility issues to assess whether contraception would not be a concern for some participants.

### Interpretation of question

Overall, participants interpreted this question as asking about contraception or help with preparing for a healthy pregnancy. Most described contraception as simply, “birth control” (focus group [FG]1, age 18–25 and FG 2, age 26–35): “just birth control, nothing else really comes to mind” (in-depth interview [IDI] 5, age 18–25). They also included abstinence and abortion in their understanding of preventing pregnancy: “everything on the spectrum…from abstinence to birth control options, to abortion” (IDI 4, age 18–25).

Participants conceptualized preparing for a healthy pregnancy in two ways: being healthy for pregnancy and fertility in terms of conception challenges and how to increase the likelihood for conception. They described service providers could offer as “folic acid” (FG 3, age 36–45) and “routine checkup[s] to make sure…the physical aspect is okay and [the pregnant person's] healthy enough to go through [their] pregnancy” (IDI 2, age 26–35).

Participants also expressed interest in discussing information about and options for getting pregnant. For example, “showing me dates of when I'm maybe ovulating. When is the best time to get pregnant for me… enough eggs and stuff like that” (IDI 2, age 26–35). Another described, “I was thinking maybe helping to track your ovulation cycle to know the sign of the best time for your body, maybe give you some tips or…reference material” (FG 6, age 36–45). Others asked for services and referrals like “in vitro” (FG 2, age 26–35) and “a test on fertility, and maybe a referral to a reproductive specialist” (FG 3, age 36–45).

Participants of all age groups interpreted the screening question as including fertility counseling, information, and services, although, younger participants discussed trying to get pregnant with respect to their own “family history” (FG 4, age 18–25) and demystifying the unknowns and myths around the ability to conceive. One described wanting to talk to their provider about “things that would help my chances to have kids… My family, actually, has genetic things going on that doesn't necessarily work before kids” (FG 1, age 18–25).

The older age group more frequently discussed fertility regarding how older maternal age could impact one's ability to conceive and potential health consequences:

When you have a child at certain age, you might have a child that's autistic or some other development disabilities, and we prepare for that as well. But having understand the risk you're taking and what is it that you can do, again, what tests I can take and be prepared. Have the discussion… “because you're getting pregnant that late, these are the problems you can have”… make sure that you understand the risks, and what are the tests that you can do if that becomes your concern. (FG 3, age 36–45)

Another described wanting their PCP to explain all options available:

One, would I still be fertile? Number two, then you get into your IVF, and surrogacy, and the whole other realm of options that may be available to me… We would have to do a rundown of what my options would be… I would have to go in for a fertility test to see if it was even possible for me to get pregnant at this point. And if it turned out that it wasn't, then we would have to do a deep dive into what other options were available… [If] it was determined that I was still able to have my own child, that would be my preference, but I wouldn't be opposed to adoption or fertility, surrogacy, fostering. (FG 3, age 36–45)

While the youngest age group did not differ significantly in their understanding of the screening question, they interpreted it as slightly more expansive compared to older groups. Beyond family planning, they perceived the screening question to refer to “test[ing] for any STDs…also cancer” (FG 4, age 18–25) and “maybe even mammograms” (FG 1, age 18–25).

### Risk and history of provider bias on services offered

Although participants interpreted the reproductive health services screening question as referring to a variety of SRH services, one assumed the provider would only refer to one type of service—preventing or planning a pregnancy—depending on where patients are in their lives and how they present to the provider. She explained:

I would assume that they're referring to planning a pregnancy. I mean, at the time of my life right now, being that I'm a little bit older and stuff, and I'm in a stable relationship, I would assume they would be talking about planning the pregnancy. (IDI 2, age 26–35)

While only one person discussed provider assumptions in the context of being asked the screening question, others across age groups raised concerns and experiences with provider bias in reproductive health care generally. For example, one Black young adult described her personal experiences with bias in contraceptive counseling where providers focused on efficacy and encouraged some options over others, rather than tailoring counseling and listening to her concerns:

It did feel rushed as we weren't talking about [contraception]. And it felt more of like a push toward a specific option… I just feel like as far as women in reproductive health, certain birth control, or certain measures might be pushed toward certain demographics… There have been women I've spoken to…that have mentioned the adverse effects of birth control. But then when you bring that up to your provider, instead of them being more relatable – reassure you that those are rare occurrences…go through different birth control options before you find the one that better suits you… – [the provider was] more information-based and provide statistics… You have your provider saying, “Oh, well, you know IUDs are the best option or…the pills might be the best option”… So, they don't bridge that gap. (IDI 4, age 18–25)

Some respondents relayed that they were not bothered by these types of interactions, and instead focused their narratives on how they are the ultimate decision-makers regarding contraceptive use and method choice.

Beyond contraception, several discussed providers expressing assumptions and gender role stereotypes about getting pregnant versus “being childless by choice” (FG 3, age 36–45). One reflected: “I didn't want to have kids when I was younger…but then they just push like… ‘you're going to want kids, you are.’ I mean…that's always what I heard… [The PCP] didn't really give you any option” (FG 2, age 26–35). Another described:

Regards to primary care doctors, you want to feel comfortable, and you want to feel non-judgmental. Sometimes if you're too young, they look at you like, “oh my gosh, you're so young, you have a kid already,” or “you're so old…you're at that stage.” You don't want them to judge you, because not everyone wants to have a kid.” (FG 6, age 36–45)

One respondent in the older group was made to feel uncomfortable by their provider for expressing interest in getting pregnant again later into adulthood: “The question that I get, “Are you done having kids?” After having three kids, it's like, “Are you done? Are you done?” I think that's a common question that makes me uncomfortable” (FG 3, age 36–45).

### Tensions accepting primary care as a provider of reproductive health services

Despite participants understanding that the screening question would be asked during a primary care visit, there was tension and pushback in accepting their PCP as the provider of reproductive health services. Some viewed Ob/Gyns as the appropriate specialists for this care, not PCPs:

I feel like that's not her specialty, the gynecologist is. That's what she does…. anything that I had that I thought was even remotely related to reproductive health, I would contact my gynecologist, make an appointment, or bring it up when I'm already there at an appointment. (FG 6, age 36–45)

Those who felt this way related their attitudes to their own experiences in primary care. For instance, in past visits, their PCPs did not have enough information to help them with their reproductive health needs and referred them to an Ob/Gyn or specialist clinic. One described:

If you talk to a PCP and tell them about that, they just gonna refer you to a Gyn, they're going to say they don't know that or like, I can't help you with that, because it has happened before with me like several times and my sister too, and it's kind of like, not annoying, but I understand that they're not in that field. (FG 2, age 26–35)

In real and hypothetical scenarios, these participants would prefer to go straight to an Ob/Gyn for their reproductive health needs because they assumed that they would ultimately be referred there. Some participants never had a PCP ask them about their reproductive health needs and would prefer to see the Ob/Gyn because they have historically filled those gaps:

I never had a primary doctor that asked me about my reproductive system… The one I have just asked me if I'm having babies or taking precaution, it was just very simple question. And I'm just like, “Yep, yep.” And he was like, “Okay, you're good.” And then he go on and getting my physical. So, for me, I still think the best way to ask for questions about my reproductive system were to go to my Ob/Gyn because she does my pap smear, she does the invasive internal checks. My primary doctor doesn't. (FG 3. age 36–45)

When discussing how PCPs can be trained to provide reproductive health care, participants were open to the possibility of receiving this care in primary care, “all in one” (FG 6, age 36–45). Some preferred primary care over Ob/Gyn because of being able to get the care they need “done in one sitting” and “avoid paying another copay and having another appointment” (FG 1, age 18–25). They also already had positive relationships with their PCPs, and cited trust and comfort as enablers for allowing them to open up about reproductive health care:

Only her. If I was to change my primary care to another person, I wouldn't feel that connection to be able to do that, really, or maybe I would, it depends on how bad the issue is. But with her in particular, I've been seeing her as my primary care since I was in middle school… I've grown up with her. My mom sees her, my grandmother sees her. So, it's like a family primary care doctor…That's the only reason why [I would receive SRH care from my PCP]. (FG 4, age 18–25)

Furthermore, some participants did not have a preference over a PCP or Ob/Gyn. Rather, what mattered was how the provider made you feel:

[If] he or she is making me feel comfortable, I wouldn't mind opening up to the doctor about reproductive health. And I wouldn't mind asking [them] questions. Versus if I felt rushed and felt like, okay, [they're] just trying to hurry me and rush through the visit. I probably would not feel comfortable asking any questions about reproductive health. (IDI 2, age 26–35)

## Discussion

Participants in our qualitative study seemed to correctly understand the open-ended reproductive health services screening question to refer to contraception and preconception care, demonstrating face validity. They also interpreted it with an expansive understanding of SRH services, including fertility and STI testing. This illustrates patients' interests in a spectrum of services they may need beyond contraception and preconception care. They are also interested in fertility, cervical and breast cancer screenings, and STI care as part of SRH services in primary care.

Although fertility can be highly complex and expensive, incorporating fertility counseling, options, and referrals in primary care may better promote reproductive justice and autonomy.^33,34^ Significant disparities in access to fertility services exist, with women of color and of lower incomes least likely to have access.^35^ Ensuring broad conversations about family planning in primary care may begin to reduce these inequities. In addition, introducing this open-ended question in primary care may enhance patient-centered care as it may allow patients to raise their SRH needs and concerns to providers when, if, and how they prefer. Given the comprehensive and holistic nature of primary care, incorporating broader SRH discussions, counseling, and services may also increase access to and continuity of care, especially for those with the greatest barriers to health care.^36^

Participants described similar experiences with implicit bias in reproductive health care, as previously identified.^34,37–40^ Most literature discusses contraceptive coercion, such as overpromoting and even forcibly providing sterilization and long-acting reversible contraception for adolescents, gender minorities, disabled communities, and people of color compared to white, middle- and upper-class women. However, participants in this study also highlighted providers' implicit assumptions that women will want to have children.

Provider bias in reproductive health may emerge due to internally held stereotypes about certain social identities and reproduction, which are also perpetuated by society, on which a provider may implicitly act.^37,41–43^ For example, gender role stereotypes about women as mothers may implicitly influence providers' language when discussing pregnancy. Rather than being told what to do, judged for their decisions, or having assumptions made about them, ultimately, patients want their providers to listen to them and collaborate when it comes to SRH care.^44^

Our group designed this open-ended screening question to increase patient-centeredness and to reduce risks of implicit bias by enabling patients to identify services they want. However, it is impossible to prevent internalized biases entirely, as evidenced by some participants' experiences, and thus, training on how to manage SRH conversations holistically is needed. To enhance autonomy and provide respectful, responsive, nonjudgmental care, health centers and medical education must incorporate initiatives to prevent implicit bias and improve patient-centered care by strengthening PCPs' skills and willingness to listen to patients' concerns and expertise and provide care tailored to individuals' needs and preferences.

While studies demonstrate that patients find receiving reproductive health services acceptable in primary care, participants in our study highlighted concerns around PCPs' SRH training and expertise, which impacted their views of primary care as an appropriate setting for reproductive health care.^21,23,45,46^ Family medicine providers are often trained in SRH care, although other PCPs, like internists and physician assistants, may receive less exposure.^47^

As such, it is critical to enhance training for all PCPs in SRH care, and to inform patients that their PCPs are trained to increase their trust and willingness to receive such services from their PCP. Integrating a routine screening question on SRH needs, such as our proposed question, may increase awareness of the availability of these services in primary care settings, which can effectively offer SRH services. In addition, SRH-themed patient education, artwork, and promotional materials in waiting and examination rooms may facilitate a safe environment for patients to raise these concerns.^48–50^ For example, some clinicians display badges, mugs, and other items in examination rooms with prompts like “Ask me about abortion care,” “pro-choice,” or “she/her pronouns” to subtly convey to patients a safe space to talk about abortion options and needs for gender expansive populations.^51^

These findings should be interpreted within the study limitations. Our sample includes those who self-identify as women, live in New York State, and are part of an online survey panel used for research recruitment; thus, findings may differ among other distinct populations.^29^ They may be a group who are interested and knowledgeable in SRH, which may have prompted their self-selected participation in this study. We did not explore the perspectives of non-English speakers, or individuals who did not self-identify as women; additional research is needed to understand a wider range of perspectives.

Furthermore, as we transitioned from focus groups to in-depth interviews to address scheduling and participation challenges for two age groups, these distinct methods may have generated different data. However, we did not find differences during analysis. The strengths of this study include the ability to recruit and engage a diverse group of women of various ages, race/ethnicities, and geographic locations within New York State, the flexible nature of our qualitative study design to respond to focus group challenges, and our rigorous iterative analysis process.

## Conclusions

This study adds to the literature evaluating a reproductive health service screening question in primary care by exploring New York women's interpretations.^21–23,27,28^ Our findings indicate face validity, in that women of reproductive age interpreted the question as it was intended. Implementing a routine, open-ended services-based screening question in primary care, such as our proposed question, may increase patients' awareness of SRH service availability, enable providers to respond to patients' SRH needs directly in primary care, and enhance patient-centered care overall. They also suggest key considerations for strengthening provider education and practice to improve patient satisfaction, awareness, and acceptability of SRH in primary care. Future research should consider testing this screening question in primary care settings to explore its effect on patient-centered SRH outcomes.

## Data Access Statement

N/A due to qualitative data.

## Abbreviations Used

Ob/Gyn: obstetrician/gynecologist
PCP: primary care provider
SRH: sexual and reproductive health
